# Supporting people with intellectual disabilities with sexuality and relationships

**DOI:** 10.1111/jar.12983

**Published:** 2022-02-24

**Authors:** Patsie Frawley, Michelle McCarthy

**Affiliations:** ^1^ Division of Education University of Waikato Hamilton New Zealand; ^2^ Tizard Centre University of Kent Canterbury UK

Supporting people with intellectual disabilities to live their lives in the fullest sense includes supporting people to be sexual, have intimate relationships and express their sexuality and sexual identities. It has been four decades since Ann Craft's pioneering work on sexuality rights of people with intellectual disabilities began framing this understanding. She challenged the dominant myths and misconceptions that saw the sexual lives of people with intellectual disabilities overlooked, restricted or pathologised. Craft's work articulated a set of rights that had not been previously named for people with intellectual disabilities that included the right to be seen as an adult, to have relationships, to be safe from abuse, to learn about sexuality and to be able to live a self‐determined sexual life that was not shaped by the views attitudes and decisions of others including staff. This ground‐breaking work has endured through the Ann Craft Trust which continues to educate, advocate and do research to progress the sexuality rights of people with intellectual disabilities. This special issue is motivated by the need to continue to advocate for these rights to be realities in the lives of people with intellectual disabilities globally.

Compiled during a period that will be remembered for the global Covid‐19 pandemic more than anything else, the special issue attracted a record number of submissions for a special issue of this journal. Manuscripts were submitted from researchers across the globe including from Spain, the Netherlands, the United Kingdom, Malta, the United States, Ireland, Canada, New Zealand, Australia and Norway. As the editors of this special issue, we thank all authors who submitted their manuscripts, including those that do not appear in this special issue, for the time and commitment they gave to this work during these ‘unprecedented times’.

This special issue was developed in a year that also saw the passing of a great researcher, advocate and spokesperson for the sexuality rights of people with intellectual disabilities, Dr. Dave Hinsburger (1952–2021). Like Ann Craft, Dave Hinsburger's work over the past two decades has shaped much of the research of the editors of this special issue. An excerpt from his book ‘I Contact: Sexuality and people with Developmental Disabilities’ (Hinsburger, 1990) says ‘The basic message of this book is that all people can love and all people can make human contact with other people’. Hinsburger goes on to say that it was through personal contact with people with intellectual disability that he learned the most about what to do and how to do it; including in relation to supporting sexuality. This is an important message to remember when reading of the articles in this special issue. Regardless of the methodologies, theoretical perspectives or areas of focus of these articles—sexuality education, ways support staff can best support sexuality rights and learning from the challenges and experiences of people with intellectual disabilities—they are all developed from some engagement with people with intellectual disabilities and all provide insights into ways ahead for continuing to support the sexual lives and sexuality rights of people with intellectual disabilities.

In this special issue of 14 articles, Ginn (2021) presents an important piece that systematically reviews the literature, paying particular attention to how it has informed practice. Like Craft and Hinsburger, Ginn is looking for ways to progress a ‘sexually just’ future for people with intellectual disabilities. She finds however that our attention has been focused by research on changing the individual rather than systemic change. This is despite a strong body of research that argues for the involvement of people with intellectual disability in shaping systemic change to progress their sexual rights. Ginn's article is a good starting point for your reading of this special issue as it challenges researchers to consider the ‘gaze’ of our work and the ways and places where we ‘project’ our findings to inform practice.

The importance of exploring and interpreting the perspectives of people with intellectual disabilities is foregrounded in a number of the papers in this special issue. Franklin et al. (2021) involved people with intellectual disabilities in their data collection and analysis of interviews with parents. They showed that despite often feeling powerless and being assumed to be incompetent, parents with intellectual disabilities were resilient and challenged the views that others held about them. They found strength in numbers, citing peer support and self‐advocacy as being key factors in helping them to overcome their marginalised status.

Santinele Martino (2021) spoke with people with intellectual disabilities about the ways in which their religious beliefs influenced their personal and sexual relationships. For some, their faith had offered them some guidance when seeking partners and forming intimate relationships. Others found their sexual expression was constrained by their religious beliefs or communities. Either way the author makes a compelling case for listening to what people with intellectual disabilities have to say about their lives and desires.

A third paper in this special issue, by McCarthy et al. (2021) argues that nowhere is it more important than in the realm of personal and intimate relationships to listen to what people with intellectual disabilities have to say. When asked in this research study, people with intellectual disabilities were very clear about the central place of intimate relationships in their lives. Life without loving relationships was described as lonely, whilst having someone to love and be loved in return was highly prized. People with intellectual disabilities are often not given opportunities to speak about their inner world, so it is all the more important that we take notice when they do.

Understanding what knowledge and awareness people with intellectual disability have about sexuality and sexual and reproductive health is the focus of three papers in this special issue. Palmer et al. (2021) look specifically at people with the genetic condition known as 22q where intellectual disability is a co‐morbidity. Importantly, this paper and those by Gil‐Llario et al. (2021a, 2021b), draw attention to the need for people with intellectual disability to receive sexuality and sexual health education, counselling and therapy that is informed by an objective assessment of their knowledge and awareness. Palmer and colleagues' paper, in particular, recommends that such assessments be used to inform ‘sex positive’ approaches in education and counselling. While much of the focus in the papers by Gil‐Llario cover technical information about the development of the two assessments, this is done to ensure that, where practice is informed by assessments, the assessments used are reliable and valid, and as these authors highlight, not limited by methodological, theoretical or practical gaps. Another focus of these articles is the place assessments can have in looking at outcomes of education, counselling and therapy by a broader group of professionals outside the disability support sector, including psychologists, sex therapists, sexual health workers, social workers and educators.

Sex education was the focus of two of the articles; Azzopardi‐Lane (2021) focussing on sex education for people with intellectual disabilities in Malta and Van Toren et al. (2021) looking at the delivery of a programme for girls with intellectual disabilities in the Netherlands. Azzopardi‐Lane's research pays particular attention to the cultural and religious context of Malta and the impact this has on the opportunities for sex education for people with intellectual disabilities, noting that this and family control over education and support opportunities is a barrier to all people with intellectual disabilities receiving sex education. An important aspect of Van Toren et al.'s study is the issue of people perceived as having ‘more ability’ being more likely to be sexually active. Those whose ‘sexual behaviour’ is determined as a problem are also more likely to receive what education is available. Van Toren et al.'s study reports the positive outcomes of the focus programme and calls for more opportunities for programmes like this one to be made available for young women with intellectual disabilities. Both papers call for this and highlight that where sex education is provided, it needs the support of families and skilled staff.

The support of staff cannot be underestimated and there are five papers in this collection which address this. Schmidt et al. (2021) and Leclerc and Morin (2021) look at staff members' perceptions of their roles when it came to providing sexuality and relationship support and information to people with intellectual disabilities. Both Oloidi et al. (2021) and Deffew et al. (2021) examined attitudes and beliefs of staff members and the effect of these on the support they did or did not provide to people with intellectual disabilities. Lunde et al.'s (2021) research in Norway, draws attention to the fact that ‘staff’ are not one homogenous group. They found there were differences in staff comfort and confidence levels to address sexuality issues based on the sex and age of staff. These are important considerations which should not be overlooked.

What all of these papers have in common is the importance of training and support for staff who undertake these skilled roles. If they do not feel confident and supported in their own right, they are unlikely to be able to provide people with intellectual disabilities with the sensitive support they are entitled to.

In conclusion, as Associate Editors, we warmly recommend the papers to readers in the hope that this new collection will make a valued contribution to the body of literature on this important topic. We have enjoyed bringing this special issue together. As we have read, reviewed, and engaged with reviewers' comments, we have considered how our collective and individual efforts in our research across the globe can continue to progress the sexuality rights of people with intellectual disabilities so they can enjoy the full expression of their personhood as equal sexual citizens.

